# Cellulose Supplementation Early in Life Ameliorates Colitis in Adult Mice

**DOI:** 10.1371/journal.pone.0056685

**Published:** 2013-02-20

**Authors:** Dorottya Nagy-Szakal, Emily B. Hollister, Ruth Ann Luna, Reka Szigeti, Nina Tatevian, C. Wayne Smith, James Versalovic, Richard Kellermayer

**Affiliations:** 1 Section of Pediatric Gastroenterology, Department of Pediatrics, Baylor College of Medicine, Houston, Texas, United States of America; 2 USDA/ARS Children's Nutrition Research Center, Houston, Texas, United States of America; 3 Department of Pathology, Baylor College of Medicine, Houston, Texas, United States of America; 4 Texas Children's Hospital, Houston, Texas, United States of America; 5 Department of Pathology and Laboratory Medicine, The University of Texas Health Science Center, Houston, Texas, United States of America; Charité, Campus Benjamin Franklin, Germany

## Abstract

Decreased consumption of dietary fibers, such as cellulose, has been proposed to promote the emergence of inflammatory bowel diseases (IBD: Crohn disease [CD] and ulcerative colitis [UC]) where intestinal microbes are recognized to play an etiologic role. However, it is not known if transient fiber consumption during critical developmental periods may prevent consecutive intestinal inflammation. The incidence of IBD peaks in young adulthood indicating that pediatric environmental exposures may be important in the etiology of this disease group. We studied the effects of transient dietary cellulose supplementation on dextran sulfate sodium (DSS) colitis susceptibility during the pediatric period in mice. Cellulose supplementation stimulated substantial shifts in the colonic mucosal microbiome. Several bacterial taxa decreased in relative abundance (e.g., Coriobacteriaceae [p = 0.001]), and other taxa increased in abundance (e.g., Peptostreptococcaceae [p = 0.008] and Clostridiaceae [p = 0.048]). Some of these shifts persisted for 10 days following the cessation of cellulose supplementation. The changes in the gut microbiome were associated with transient trophic and anticolitic effects 10 days following the cessation of a cellulose-enriched diet, but these changes diminished by 40 days following reversal to a low cellulose diet. These findings emphasize the transient protective effect of dietary cellulose in the mammalian large bowel and highlight the potential role of dietary fibers in amelioration of intestinal inflammation.

## Introduction

Inflammatory bowel disease (IBD), including Crohn disease (CD) and ulcerative colitis (UC), have been recognized as disorders whereby environmentally sensitive developmental factors may play an important etiologic role [1]. The peak incidence of IBD is in young adulthood. Therefore, there is a prolonged developmental period from conception to young adulthood for environmental influences to critically impact biological systems relevant for IBD pathogenesis [2]. Important elements of IBD pathology are thought to be the intestinal microbiome, the gut mucosa, and the mucosa associated immune system [1], [3]. We have recently shown in a mouse model that maternal supplementation of 4 micronutrients could significantly modify offspring colitis susceptibility in association with colonic mucosal gene expression and microbiome alterations [4], [5]. However, the same diet did not induce obvious phenotype changes with respect to intestinal inflammation when given during pediatric development. These findings underscored that pertinent nutritional factors can exert persistent colitis modifying effects during critical periods of mammalian maturation.

As for IBD, the decreased consumption of dietary fibers has been recognized to potentially play an etiologic role [6]. The rising incidence of IBD in the developed world associating with a decrease in dietary fiber intake was emphasized by Burkitt during the early 70's [7]. In agreement with this observation, a recent study highlighted the importance of different nutritional habits and gut microbial diversity in children from two different continents and cultures (European and a rural African village) [8]. The authors called the attention to the reduction of fiber intake and the associated decrease of microbial richness in European children, which may be relevant in respect to gastrointestinal diseases. Indirect epidemiologic data supports these conclusions where high fiber and fruit consumption are associated with a decreased risk of CD and high vegetable intake is associated with a decreased risk of UC [6].

Cellulose is an insoluble fiber and an abundant component of a vegetarian diet since it is present in most plant tissues [9]. It has a proliferative effect on the colon, mostly in the distal areas in rodent models [10]. The colonic trophic effects of cellulose do not depend on microbial fermentation [11], as opposed to the case of other fibers, such as guar gum [12]. Although cellulose may be fermented in the colon, the secondary production of short-chain fatty acids (SCFAs) is limited [13]. Despite the limited fermentation of cellulose, it has been shown to substantially modify colonic microbial composition [14]. These results implicate it as a potential prebiotic (non-digestible carbohydrate that favors the growth of desirable microflora in the large bowel). Since cellulose is a major component of vegetable and fruit fibers, the indirect nutritional epidemiologic data already discussed [6], [8] would support it as an important factor in IBD pathogenesis. However, the effect of cellulose on mammalian colonic mucosal microbiome (which may be more relevant for IBD pathogenesis than luminal bacteria [15], [16]), or colitis susceptibility has not yet been investigated especially from the developmental origins perspective.

Therefore, in this study, we examined the direct and prolonged effects of pediatric cellulose supplementation on large intestinal growth, chemically induced colitis susceptibility, and colonic mucosal microbial community composition in mice.

## Materials and Methods

### Animals, diets and experimental design

Our initial observations were made on C57BL/6J male mice (Jackson Laboratories, Bar Harbor, ME, USA) receiving (regular chow [12.5% dietary fiber]; 2920X, Harlan-Teklad, Madison, WI, USA) or synthetic low cellulose (2.5% cellulose, LC; #102460, Dyets Inc., Bethlehem, PA, USA) from 30 to 90 days of age. In the consecutive experiments, 21 day old (postnatal days 21, P21) C57BL/6J male mice were provided free access to regular chow (12.5% dietary fiber) within the same room of our animal facility for 9 days. At P30, the mice were randomly allocated to receive synthetic low cellulose or high cellulose (12.5% cellulose, HC; #102532, Dyets Inc., Bethlehem, PA, USA) diet for 50 days (Table S1). Conventional chows regularly contain ∼12.5% fiber (including cellulose). At P80, the HC animals were reversed to control (low cellulose) diet for 10 (HCR10 = P90), or 40 days (HCR40 = P120) (Figure S1). For the microbiome studies, 10 animals in two cages were used for each dietary group. Those were allocated to receive either low cellulose (control, LC, 10 animals in 2 cages), or high cellulose diets (HC, 10 animals in 2 cages). At P80, 2 animals from each cage were crossed between the groups to eliminate cage bias. Five control animals at P80 and 5 at P90 were euthanized for tissue collection. There was no significant separation between these groups based upon a principle coordinates analysis (PCoA) of *16S rRNA* sequence information; therefore, these were grouped together as a single control. As for the high cellulose group, 2 animals were crossed between the two cages at P80. One cage was continued on high cellulose diet until P90 (HC), and the other cage was reversed to the control diet (low cellulose, LC) until P90 (HCR10), when those were euthanized for tissue collection. The discovery cohorts included 3-3 mice in two independent studies (P90 LC and HC; and P90 LC and HCR10) with the same experimental conditions. All applicable institutional and governmental regulations concerning the ethical use of animals were followed. The protocol was approved by the Institutional Animal Care and Use Committee of Baylor College of Medicine.

### Dextran sulfate sodium exposure

Susceptibility to colitis was tested by administering 2% (wt/vol) (for the synthetic chow experiments) or 3% (for the original regular chow vs. synthetic chow observations) dextran sulfate sodium (DSS; MW = 36000–50000, MP Biomedicals, LLC, Solon, OH, USA) in the drinking water at P90 or P120 *ad libitum* for 5 days followed by regular water for an additional 9 days (Figure S1). DSS of this molecular weight induces diffuse colitis from cecum to distal large bowel [17]. The animals were weighed daily and colonic length measurements were performed at the end of the experiments following CO_2_ asphyxiation. Weight loss during DSS administration in mice has been shown to correlate well with molecular and histological outcome measures of colitis severity [2], [18], [19]. Therefore, we decided to follow weight loss, colon length, and histological severity of intestinal inflammation as the primary outcomes measuring colitis severity in our DSS experiments.

### Tissue collection and histological analysis

At the end of the feeding periods, mice were sacrificed by CO_2_ asphyxiation between 11:00 AM and 2:00 PM without any previous food restriction. The colons were placed on ice, transected longitudinally, cleansed from feces, washed with ice cold normal saline, followed by the collection of colonic mucosa with a microscope slide [20] (excluding the cecum). The mucosal scrapings were flash frozen on dry ice, and stored at −80°C as earlier described [2].

For histological analysis, additional colonic samples (5 LC and 5 HCR10) were transected and processed for standard hematoxylin-eosin staining following fixation in 10% formaldehyde. Histological severity of intestinal inflammation was determined by a blinded pathologist based upon tissue damage grade and the presence of inflammatory cells. [21]

Morphometric analyses (crypt length, surface area) were performed with Olympus DP2-BSW program on transected proximal (post-cecal) colonic specimens from P90 LC and HC animals.

### DNA extraction and analysis of microbiome

Colonic mucosal samples were submitted to the Texas Children's Microbiome Center (Texas Children's Hospital, Houston, TX, USA) for DNA extraction and sequencing. Community DNA was extracted from each specimen using the PowerSoil DNA isolation kit (Mo Bio Laboratories, Carlsbad, CA, USA), following the Human Microbiome Project modifications [22] to the manufacturer's instructions. The resulting DNA was quantitated using both a NanoDrop-1000 spectrophotometer (NanoDrop, Wilmington, DE, USA) and Qubit fluorometer (Life Technologies Corporation, Carlsbad, CA, USA). Barcoded universal primers 357F (5′-CCTACGGGAGGCAGCAG-3′) and 926R (5′-CCGTCAATTCMTTTRAGT-3′) were utilized to amplify the V3-V5 region of the *16S rRNA* gene. The discovery study utilized a different set of PCR primers (28F [5′-GAGTTTGATCNTGGCTCAG-3′] and 519R [5′-GTNTTACNGCGGCKGCTG-3′] amplifying the V1–V3 region of the *16S rRNA*). Each library construct was then processed and purified for 454 sequencing. Sequencing was performed on the Roche GS FLX 454 sequencer (454 Life Sciences, Branford, CT, USA).

Sequence data were parsed by barcode and quality filtered using QIIME (version 1.3.0) [23], as implemented in the Genboree Microbiome Toolset [24]. Sequences shorter than 200 bp length, having average quality scores less than 20, harboring ambiguous base calls, or having mismatches to their barcode or sequencing primer were excluded from further analysis. Both the barcodes and sequencing primers were trimmed away, and the remaining sequences from the control (LC), HC and HCR10 treatments were pooled and assigned to operational taxonomic units (OTUs) at a similarity cut off of 97% using Cd-hit [25]. The data set was screened for potential chimeras using the ChimeraSlayer algorithm [26], and all potential chimeras were excluded from downstream analysis. Identities were assigned to each OTU using the Ribosomal Database Project Classifier [27].

Prior to the calculation of diversity metrics or comparison across treatments, the sequence libraries were randomly subsampled to achieve even sampling depth (subsampled depth = 3634 sequences per library). All results presented here were determined using our subsampled sequence libraries. Community similarity was evaluated across treatments among treatments using principle coordinates analysis (PCoA) of OTU data. PCoA plots were generated using weighted Unifrac distances, as calculated in QIIME.

Sequence libraries were also evaluated for potential differences in composition using two-tailed Mann-Whitney U-tests and false discovery rate correction for multiple comparisons in Genespring GX (version 12.0) (Agilent Technologies, Santa Clara, CA, USA). A stratified approach was used, first identifying OTUs that differed significantly with respect to the LC vs. HC treatment, then exploring these differences further in the context of the HCR10 treatment, with the goal of identifying changes that may be transient (i.e. quick return to LC levels) or longer-lasting (i.e. continuing to resemble the HC condition).

The sequences generated for this project were deposited in the NCBI Sequence Read Archive (SRA) under project accession SRP013763.

### Additional statistical analysis

Unpaired two tailed t-tests, and two tailed non-parametric Mann-Whitney U-tests were utilized in the group comparisons where statistical significance was declared at p<0.05. Error bars represent standard error of the mean (SEM).

## Results

### Increased severity of DSS colitis on a synthetic, low fiber diet

**Figure 1:**
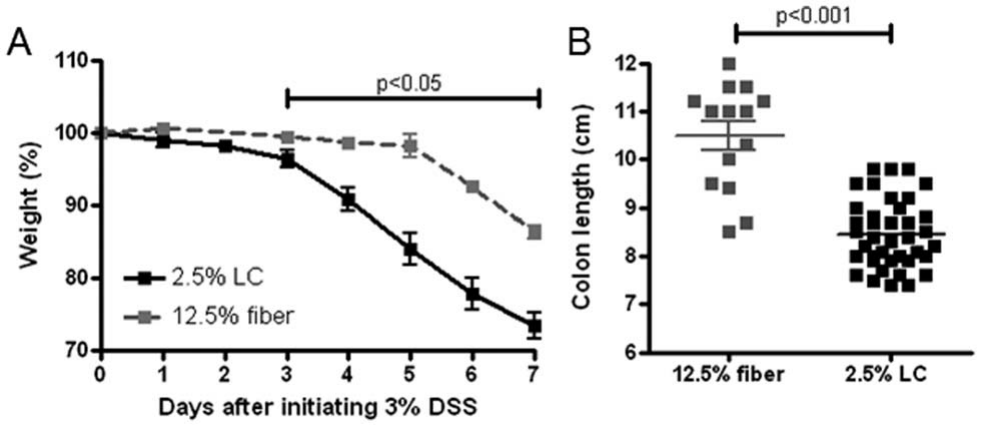
Increased severity of colitis and decreased colonic lengths in P90 mice fed by a synthetic diet [low cellulose (2.5%, LC) as fiber] compared to regular chow (12.5% dietary fiber). Upon 3% DSS challenge (Figure 1A, n = 8–10), the animals on the LC diet lost significantly more weight than on regular chow. On the seventh day, the experiment had to be terminated for severe weight loss in the LC group. In independent experiments where the mice did not receive DSS (Figure 1B, n = 14–40), colonic lengths of mice fed the synthetic diet were significantly shorter than in animals fed regular (12.5% fiber) chow.

Chemical colitis in C57BL/6J mice is usually induced by transient exposure to 3–5% DSS [17], [28]. When we initiated 3% DSS treatment in animals that received a synthetic, low cellulose (2.5% cellulose, LC) diet, they lost excessive weight necessitating early euthanasia (Figure 1A). Therefore, mice on the LC diet had significantly increased susceptibility to chemically induced colitis compared to animals on regular chow (12.5% dietary fiber). Based on our findings, we utilized 2% DSS treatment to induce colitis in our further experiments involving low cellulose diets.

Upon comparing large intestinal lengths between mice receiving regular chow and our synthetic (LC) diet, we recognized that colons were significantly shorter on the artificial diet (Figure 1B) without any previous exposures (such as DSS). Since dietary fibers and cellulose specifically have been shown to promote intestinal lengthening (trophic effects) in rodents [10], [29], we postulated that the low cellulose content of the synthetic diet may have been responsible for the observed shorter baseline colon lengths (leading to smaller colonic surface area) and increased susceptibility to DSS colitis (secondary to increased DSS to relative colonic surface, i.e. higher concentrations of the chemical irritant at the mucosa).

### Transient trophic and anticolitic effects of cellulose supplementation

**Figure 2:**
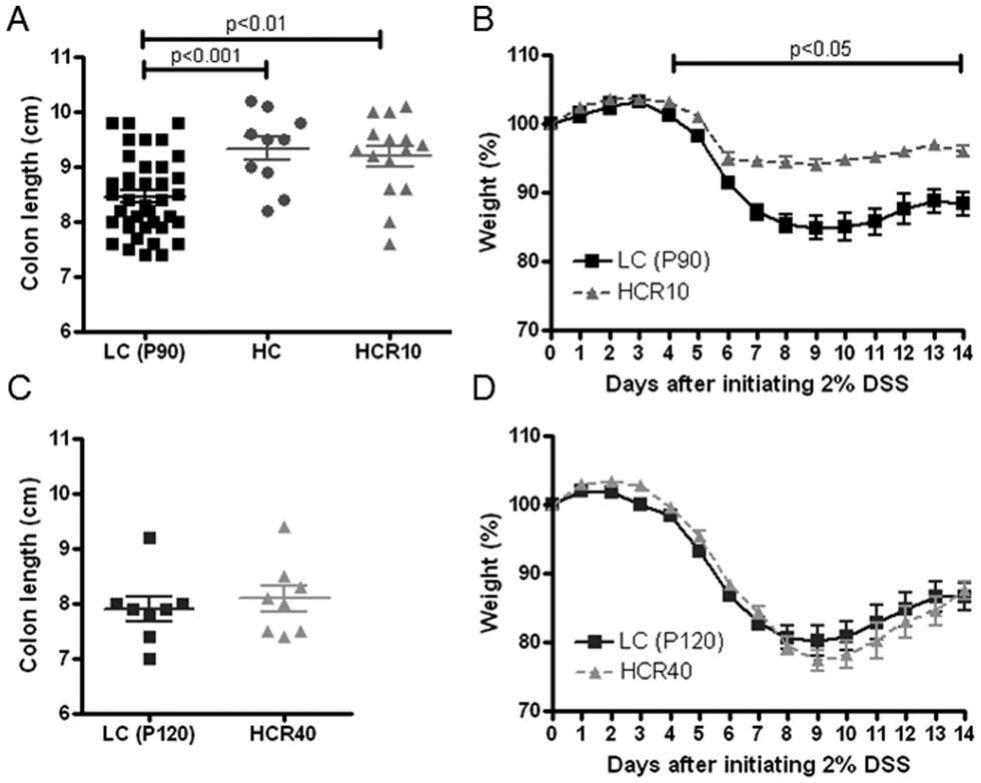
Transient colonic trophic effect and protection against dextran sulfate sodium (DSS) colitis upon cellulose supplementation. **A.** Colons were significantly longer in animals receiving 12.5% cellulose supplemented diet (HC). This trophic effect persisted after 10 days of reversal (HCR10) from the 12.5% cellulose to 2.5% cellulose diet (LC P90) [n = 40-10-15]. **B.** Other mice were exposed to 2% DSS (as opposed to 3% in Figure 1, due to the increased sensitivity of mice to DSS on the low cellulose diet) in their drinking water at P90 for 5 days then received regular water. Mice reversed for 10 days (HCR10) from the high cellulose (12.5%) diet were protected (less weight loss) against DSS compared to controls (LC P90) [n = 7–10 per group]. The histological score confirmed our results: high cellulose supplemented group had decreased severity of colitis upon DSS challenge (See Figure S2). **C.** The cellulose supplementation induced trophic effect was lost after 40 days of reversal (HCR40) from the 12.5% cellulose to 2.5% cellulose diet (LC P120) [n = 8-8]. **D.** Colitis susceptibility was also similar between the 40 day reversal (HCR40) and control groups (LC P120) [n = 7–10 per group].

Dietary fibers have been observed to decrease colitis severity in acute and chronic rodent models [30], [31], [32]. However, the means by which this effect is accomplished, and whether all fiber types have such anti-inflammatory properties, remains questionable. We were particularly interested if dietary cellulose supplementation by itself could decrease DSS colitis severity and how persistent such effects may be. Consequently, the cellulose content of our synthetic diet was selectively increased (high cellulose, HC; Table S1) and fed to the animals during the pediatric period (P30–P80 [33]). Thereafter, the mice were reversed back to the LC diet for 10 (high cellulose reversed 10 days: HCR10 = P90 total), or 40 (HCR40 = P120 total) days to examine the persistence of any anticolitic effect induced by pediatric cellulose supplementation. The pediatric period is highly relevant with respect to the nutritional and developmental origins of IBD since the disorders most commonly present in young adulthood [2]. Colons were significantly longer in mice fed a high cellulose diet even after 10 days of reversal compared to controls (Figure 2A). Remarkably, the large intestines were similar in length compared to control following 40 days of reversal (Figure 2C). The severity of DSS (2%) colitis correlated inversely with baseline colonic lengths in the feeding groups. Mice reversed for 10 days from the high cellulose diet (HCR10) were protected compared to the controls (LC) (Figure 2B). Colitis susceptibility was similar between the 40 day reversal (HCR40) and LC groups (Figure 2D). The severity of histological inflammation upon DSS challenge also decreased (p = 0.016) in the HC dietary group compared to controls (LC) supporting the findings on weight loss and colonic shortening (Figure S2).

**Figure 3:**
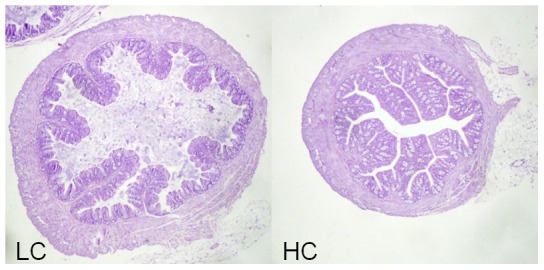
Transected proximal colons from LC (left) and HC (right) mice at P90. Note the significantly (p<0.001; 7–8 measurements, average length 1180 µm vs. 393 µm) elongated crypts in HC compared LC. The surface area increase secondary to this morhological change was calculated to be 20% in HC compared to LC (magnification ×40).

Morphometric analyses on representative proximal colonic samples from HC and LC (Figure 2A) mice showed that HC leads to significant (p<0.001) elongation of crypts leading to a 20% increase in transectional surface area (Figure 3). The overall average colonic surface area increase upon cellulose supplementation was calculated to be around 30%.

### Microbial diversity and composition changes following cellulose supplementation

Mucosa associated microbes may be more important in gut homeostasis and inflammation than luminal ones [16]. The effect of different fibers on the metabolic activity and composition of the gut microbiome was observed both in fecal samples and in *in vitro* models [8], [34], [35]. Nevertheless, the consequence of dietary fiber (including cellulose) supplementation on the colonic mucosal microbiome in mammals has not been studied, especially with high-throughput methodologies. Therefore, we examined if dietary cellulose supplementation may affect the colonic mucosal microbiome. The persistence of effects on the gut microbiome 10 days following a decrease in cellulose consumption was also studied in a discovery and a validation cohort.

**Figure 4:**
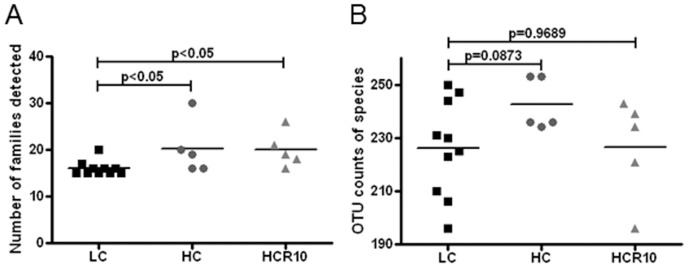
Cellulose related increase in microbial richness. **A.** High cellulose (12.5%, HC) diet stimulated a significantly increased number of detectable families compared to low cellulose (2.5%, LC). This separation persisted 10 days following reversal (HCR10). **B.** The OTU counts of species were slightly increased in the HC group compared to LC, but not in the reversal group (HCR10). p values represent two tailed non-paired T test.

A variety of microbial taxa were detected within the colonic mucosa of mice on the HC, LC, and HCR10 diets. Although several could be identified to genus level, the majority could not be identified confidently beyond the family level. Several bacterial families were detected in increased quantities in the colonic mucosa of mice on the HC diet compared to LC (Figure 4A). This fiber-dependent augmentation of microbial richness was persistent up to 10 days of reversal to the low cellulose diet (HCR10). A tendency toward increased OTU richness was also observed in the HC group compared to LC, but was diminished by 10 days reversal (Figure 4B). The HC OTU richness was on average 242 OTUs, while HCR10 and LC had a same average of 226.

**Figure 5:**
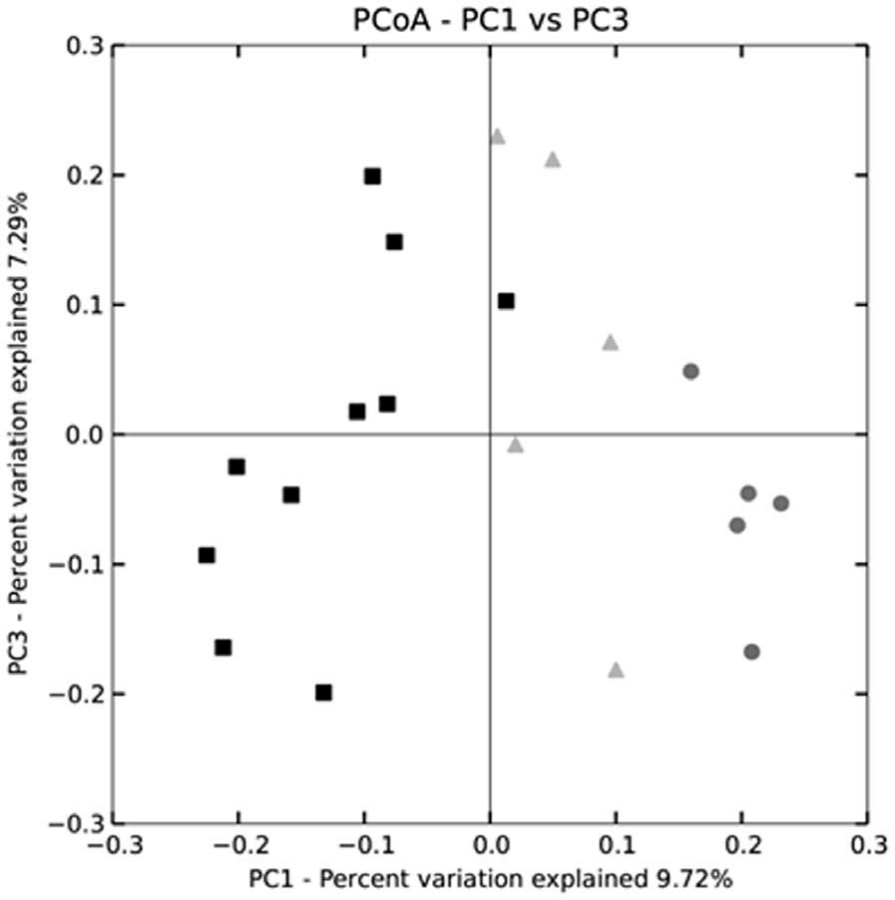
Cellulose induced shifts in the composition of the gut microbiome. Principle coordinates analysis (PCoA) shows separation of the colonic mucosal microbiome on high cellulose diet (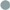
high cellulose [12.5%]; n = 5). This separation decreased by 10 days of reversal (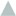
10 days reversal from high cellulose diet [HCR10]; n = 5) compared to controls (▪control [2.5% cellulose] diet; n = 10). Individual animals did not show separation depending on cage origin.

**Figure 6:**
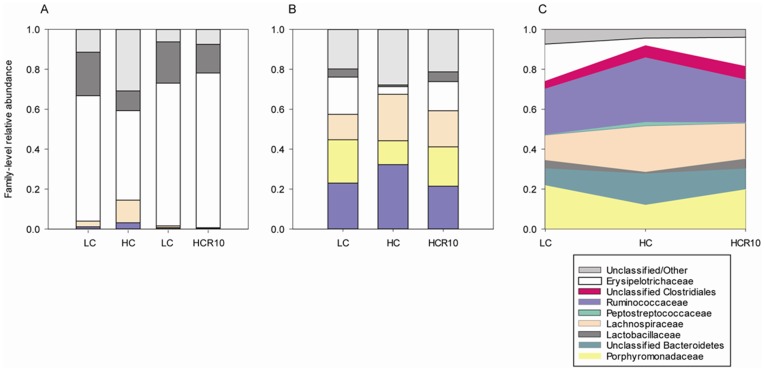
A. Relative abundance of the most common families within the colonic mucosal samples of the discovery group. Altered relative abundance of bacterial families was presented upon high cellulose (HC) diet compared to control (Low cellulose, LC). This alteration diminished following 10 day reversal (HCR10). **B. Relative abundance of the five most common** (and the unclassified) **families within the colonic mucosal samples.** The HC group is more different from the LC than the 10 day reversal dietary group (HCR10). **C. Abundance variation in the 10 most abundant bacterial families between the dietary groups.** A trend for reversal in abundance can be seen in most of the taxa by 10 days of reversal (HCR10) from the high cellulose diet (HC) in relationship to the low cellulose (LC) group.

Principle coordinates analysis (PCoA) showed a distinct separation between the HC and LC group communities (Figure 5). HCR10 samples clustered between the LC and the HC microbiome, indicating partial reversal of the cellulose induced separation following 10 days on low cellulose diet. Cage effects did not influence community separation within the groups (see Materials and Methods for details). The most abundant family level taxon changes mirrored the PCoA results (Figure 6), in which the HC and LC groups appeared to be distinctly different from one another, and the HCR10 group was intermediate to the two others, but perhaps closer to the LC group.

**Figure 7:**
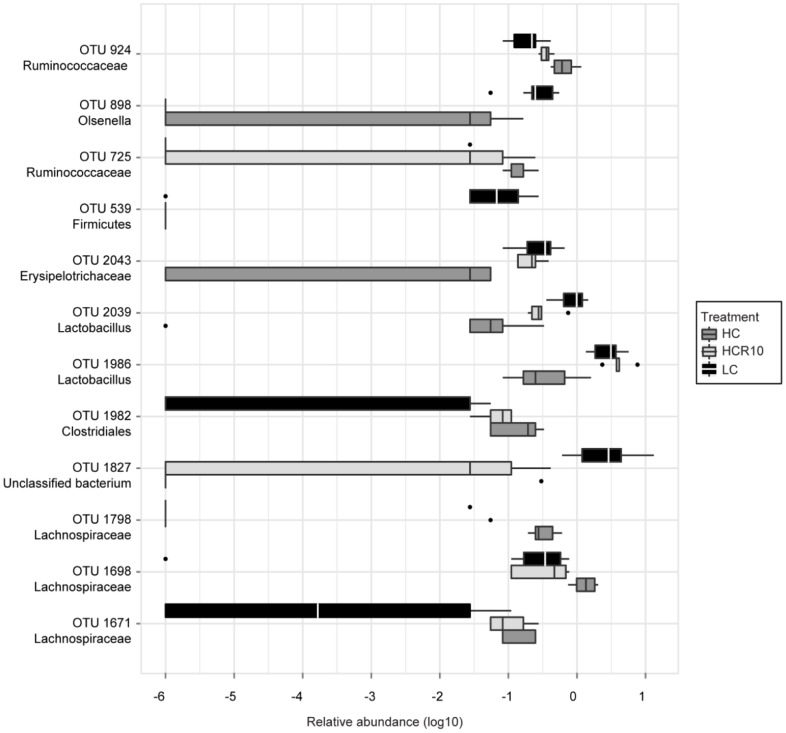
Boxplots depicting shifts in OTU abundance following diet reversal. A stratified approach was used to identify transient changes in OTUs. Each of the OTUs depicted differed significantly between the HC and the LC treatment, as well as between the HCR10 and the HC groups, or between the HCR10 and the LC groups.

Two phyla (Actinobacteria and Tenericutes) were significant increased and diminished in abundance, respectively in the HC group compared to control (LC). Only members of the phylum Actinobacteria showed persistently decreased abundance after 10 days of reversal (Table 1). An example of this can be seen in Figure 7, where an Olsenella-like OTU (i.e. OTU 898) demonstrated significantly decreased abundance in the HC and HCR10 groups relative to controls (LC).

**Table 1:** The effects of cellulose supplementation on colonic mucosa associated bacterial phyla and families.

				HC vs. LC		HCR10 vs. LC	
	LC	HC	HCR10	p	U	p	U
**PHYLUM**							
**Actinobacteria**	0.365988	0.115575	0.088057	0.010551	0.0193	0.002612	0.0007
**FAMILY**							
**Coriobacteriaceae**	0.365988	0.071547	0.055036	0.002391	0.0007	0.001153	0.0007
**Peptostreptococcaceae**	0	1.733627	0.093561	2.77E-08	na	0.008005	na
**Clostridiaceae**	0.156852	1.056687	1.172262	0.001377	0.0013	0.047661	ns

Actinobacteria was significantly modified in abundance secondary to increased amounts of dietary cellulose (HC: high [12.5%] cellulose vs. LC: low [2.5%] cellulose), and separated at 10 days of reversal (HCR10) from the LC group as well. There were three families (Coriobacteriaceae, Peptostreptococcaceae and Clostridiaceae) with significant and persistent abundance difference on HC and HCR10 compared to controls (LC) following the temporary cellulose supplementation. p values represent two tailed non-paired T test, U values represent two tailed non-parametric Mann-Whitney U-test (ns: not significant, na: not applicable).

Our discovery cohort indicated a decrease in the family level abundance differences between the HC and the LC groups following 10 days of reversal to the LC diet from HC (Table S2A).The validation experiments conformed these results (Table S2B). There were 10 distinguishable families with significant abundance difference between the HC and the LC groups. This number decreased to 6 families by 10 days of reversal (HCR10) with overall higher p values than in the HC-LC comparisons (data not shown). Persistent family abundance differences in the HC and the HCR10 groups, when compared to the LC group, were in Coriobacteriaceae (decrease), Peptostreptococcaceae and Clostridiaceae (increase) (Table 1).

## Discussion

Murine pediatric cellulose supplementation induces transient trophic, anticolitic and microbiome-modifying effects according to this study. The cellulose-stimulated lengthening of the colons persisted for 10 days, which was associated with protection against DSS. Additionally, we found that the cellulose-dependent trophic and anticolitic effects were transient by being lost 40 days after reversal of cellulose supplementation. This later result highlights the remarkable capacity for the large bowel towards anatomical reconstitution in mammals during young adulthood (between P90 and P120). The means by which DSS provokes colitis is still of debate, but recent studies indicate that it induces hyperosmotic stress leading to NF-κB activation through the posttranslational methylation of protein phosphatase 2A [36]. Regardless, toxin-induced colitis is bound to depend on the toxin-amount/mucosal-surface-area ratio. Lengthier colons with larger mucosal surface in the same species are likely to be less vulnerable to the toxin if the animal consumes the same amount. The average surface area increase induced by cellulose supplementation in this dietary model was calculated to be around 30%. Consequently, our study suggests that cellulose supplementation during animal development induces growth of the large intestine, thereby leading to an increased mucosal surface that decreases the toxin to surface-area ratio and ultimately provides protection against noxious insults.

Dietary fibers have been shown to modify both microbial composition and colitis susceptibility in mammals. Therefore, we studied the consequences of dietary cellulose on the colonic mucosal microbiome composition as well. Cellulose supplementation induced a significant microbiome separation by PCoA. Cluster differences were less pronounced following 10 days of reversal to low cellulose, but those were not diminished completely. This observation was true for the increased richness of the microbiome during high cellulose supplementation and 10 days of reversal as well. These findings support the presumption of De Filippo and colleagues [8] that increased dietary fiber consumption may be an important reason for the enhanced stool microbiome richness in rural African children compared to European children. Given that the animals in this work were maintained in the same facility room and even transferred between cages, our results also indicate that cellulose supplementation dynamically increases the inter-species tolerance within mammalian commensal microbiota thereby inducing increased gut microbial richness within the same geographic environment. This finding contradicts the conclusions of De Filippo and colleagues [8], who suggested that a fiber-rich diet provides means to maintain overall enteral bacterial species richness by sustaining a diverse microbial environment within a geographical location. Rather, cellulose consumption (i.e. fiber-rich diet) can increase intestinal microbiota richness within the same microbial environment and can readily support mucosal protection.

At the phylum level, Actinobacteria decreased persistently during high cellulose supplementation and following 10 days reversal. This finding coincides with observations in *TLR2−/−* mice, which can be more susceptible to colitis than WT [28]. Actinobacteria were present in greater abundance in colonic mucosa of *TLR2−/−* mice similar to human studies on IBD associated microbiome [37], [38]. The decreased abundance of Actinobacteria in our current work was associated with a milder form of DSS colitis, supporting a potential colitic effect for this phylum in mammals.

Three families were modified in a prolonged fashion (in both HC and HCR10) upon cellulose supplementation: Coriobacteriaceae (a family within the phylum Actinobacteria) decreased, and Peptostreptococcaceae and Clostridiaceae increased compared to controls. The abundance of Peptostreptococcaceae and Clostridiaceae increased in human studies examining biopsies and stool samples from chronic pouchitis patients [39], [40]. Intestinal mucosal ulceration and the severity of chronic pouchitis were associated with increased numbers of different species within Peptostreptococcaceae and Clostridiaceae. Based on these findings, it is difficult to interpret the significance of the changes affecting these taxa in our experiments where pre-inflammation (no DSS exposure) microbiome composition was examined. Similar debates about microbiome associations of IBD (i.e. primary change, or secondary effect of inflammation) have been raised [41].

The overall decline of family level composition variation upon 10-day reversal from high cellulose diet may indicate that the persisting gut morphological changes (i.e. longer colons with larger mucosal surface) may be more important towards colitis protection than the remaining shifts in community composition of the colon in this feeding model. This conclusion is supported by a recent publication highlighting the importance of Allobaculum (a member of the Erysipelotrichaceae) and Paludibacter (a member of the Porphyromonadaceae) as key contributors to DSS colitis sensitivity [42]. We detected reduced abundances of Erysipelotrichaceae and Porphyromonadaceae on high cellulose diet (Figure 6), which could contribute to colitis protection (with less severe colitis). However, most of these changes diminished after 10 days reversal (both in our discovery and validation experiments) suggesting a less important influence of the microbiome on the colitis of the HCR10 mice than their longer large intestinal phenotype.

This work includes the first in depth analysis of effects of dietary cellulose supplementation on the composition of the mammalian colonic mucosal microbiome. The results indicate a transient effect of this nutritional intervention even following a prolonged high cellulose exposure. The developmental origins-based nutritional approach (testing the prolonged effects of a temporary nutritional exposure during the pediatric period) demonstrated that the colonic trophic effects of cellulose are also transient. These findings underscore the significant constitutional and morphological adaptive capacity of the colonic microbiome and the large intestine, respectively. Temporary microbial and intestinal changes were associated with transient protection against experimental colitis in mice. Such massive adaptive capabilities demonstrate the dynamic responsiveness of the mammalian gut [43] towards luminal physical/compositional changes. The findings of this work may have implications for establishing dietary guidance for the promotion of large intestinal health in humans and the need for sustained dietary interventions for lasting effects on the intestinal microbiome and intestinal resilience.

## Supporting Information

Figure S1 **Schematic description of the feeding protocols.** **A:** 12.5% high cellulose (HC) group without reversal for the purposes of colonic length and microbiota analysis. **B:** 2.5% low cellulose (LC) group. **C:** 10 day reversal group (HCR10). **D:** 40 day reversal group (HCR40). For the B, C and D groups DSS was administered at postnatal day 90 (P90) and P120, respectively. (DSS: dextran sulfate sodium; R10 and R40: 10 or 40 days of reversal following high cellulose diet; P21/30/80/90/120, 30/80/90/120 days postnatal age).
(TIF)

Figure S2 **Histological severity of colitis in a validation experimental group.** Colitis severity was significantly (U test p = 0.016) decreased in the 12.5% high cellulose (HCR10) group compared to controls (low cellulose, LC). N = 5. Cellulose supplementation was given between postnatal day 30 to 80, then DSS treatment was administered for 5 days. We did follow-up altogether with the DSS-treatment for 14 days.
(TIF)

Table S1 **The composition of the synthetic low cellulose (LC: 2.5% cellulose) and the high cellulose (HC: 12.5% cellulose) diets.**
(DOC)

Table S2 **The effects of cellulose supplementation on colonic mucosa associated bacterial families.** **A.** Bacterial families differing with respect to cellulose supplementation [HC: high cellulose vs. LC: low cellulose, control; and 10 days of reversal (HCR10) vs. LC] in our discovery groups. Following 10 days reversal, the microbiome separation decreased between the cellulose supplemented and control groups (7 families differed in HC vs. LC; 4 families differed in HFR10 vs. LC). **B.** Bacterial families differing in HC and HCR10 group compared to controls in the validation cohort. There were 10 families with significant abundance difference on HC compared to controls. This number decreased to 6 families by 10 days reversal (HCR10 vs. LC). Therefore, similar decreases in differing family numbers occurred in both the discovery and the validation cohorts. Furthermore, on high cellulose diet, bacterial family (Peptostreptococcaceae, Lachnospiraceae, Clostridiaceae increased; Lactobacillaceae and Erysipelotrichaceae decreased in HC group compared to LC) abundances changed the same way in both the discovery and experimental groups (bold). p values represent two tailed non-paired T test, U values represent two tailed non-parametric Mann-Whitney U-test (ns: not significant, na: not applicable).
(DOC)
