# Supplementary figures and images for: Cellulose Supplementation Early in Life Ameliorates Colitis in Adult Mice
Source: PLoS One. 2013 Feb 20;8(2):e56685. doi: 10.1371/journal.pone.0056685 (PMC3577696; doi:10.1371/journal.pone.0056685)

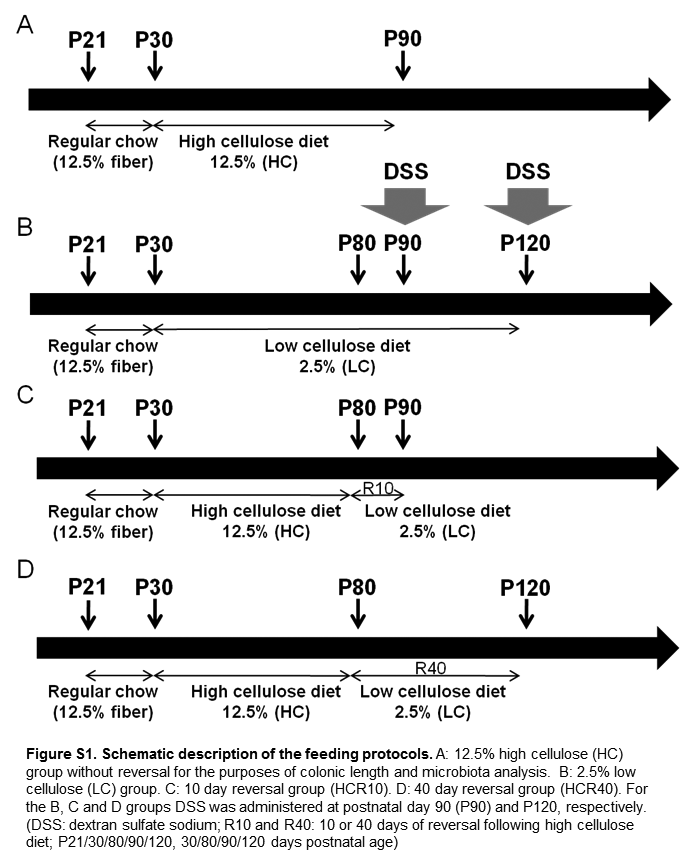

Supplement: Figure S1 — Schematic description of the feeding protocols. A: 12.5% high cellulose (HC) group without reversal for the purposes of colonic length and microbiota analysis. B: 2.5% low cellulose (LC) group. C: 10 day reversal group (HCR10). D: 40 day reversal group (HCR40). For the B, C and D groups DSS was administered at postnatal day 90 (P90) and P120, respectively. (DSS: dextran sulfate sodium; R10 and R40: 10 or 40 days of reversal following high cellulose diet; P21/30/80/90/120, 30/80/90/120 days postnatal age). (TIF) [file pone.0056685.s001.tif]

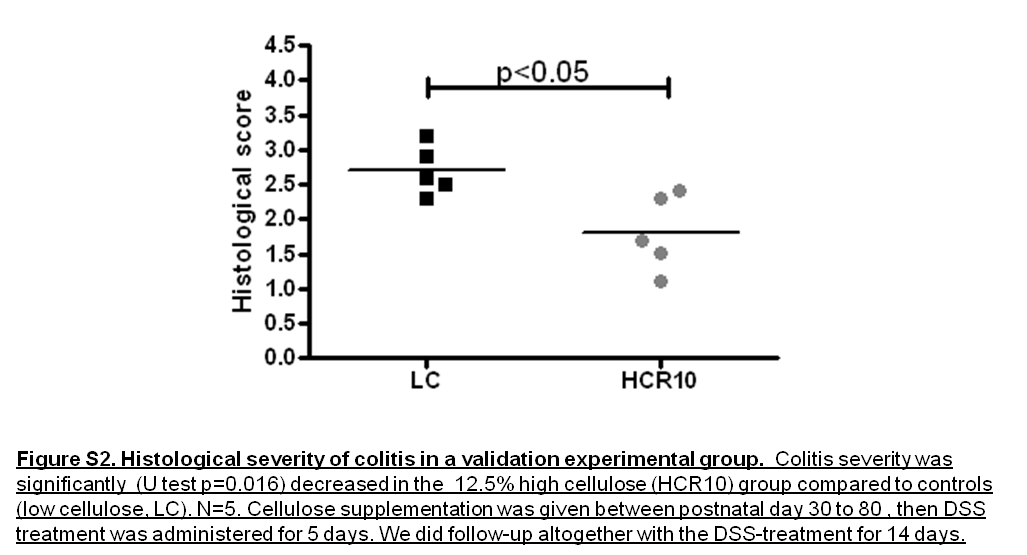

Supplement: Figure S2 — Histological severity of colitis in a validation experimental group. Colitis severity was significantly (U test p = 0.016) decreased in the 12.5% high cellulose (HCR10) group compared to controls (low cellulose, LC). N = 5. Cellulose supplementation was given between postnatal day 30 to 80, then DSS treatment was administered for 5 days. We did follow-up altogether with the DSS-treatment for 14 days. (TIF) [file pone.0056685.s002.tif]
