# Supplementary material for: Cellulose Supplementation Early in Life Ameliorates Colitis in Adult Mice
Source: PLoS One. 2013 Feb 20;8(2):e56685. doi: 10.1371/journal.pone.0056685 (PMC3577696; doi:10.1371/journal.pone.0056685)
Supplement: Table S1 — The composition of the synthetic low cellulose (LC: 2.5% cellulose) and the high cellulose (HC: 12.5% cellulose) diets. (DOC) [file pone.0056685.s003.doc]

**Table S1. The composition of the synthetic low cellulose (LC: 2.5% cellulose) and the high cellulose (HC: 12.5% cellulose) diets.**

|  |  | **LC** | | **HC** | **LC** | **HC** | |
| --- | --- | --- | --- | --- | --- | --- | --- |
| **Ingredient** | **kcal/gm** | **grams/kg** | | | **kcal/kg** | | |
| **Casein** | **3.58** | **200** | | | **716** | | |
| **DL-Methionine** | **4** | **3** | | | **12** | | |
| **Sucrose** | **4** | **341.46** | | | **1366** | | |
| **Cornstarch** | **3.6** | **242.5** | **172.5** | | **873** | | **621** |
| **Dyetrose** | **3.8** | **90** | **60** | | **342** | | **228** |
| **Corn Oil** | **9** | **51** | | | **459** | | |
| **Cellulose** | **0** | **25** | **125** | | **0** | | |
| **Mineral Mix**  **#200000** | **0.47** | **35** | | | **16.45** | | |
| **Vitamin Mix**  **#300050** | **3.92** | **10** | | | **39.2** | | |
| **Choline Bitartrate** | **0** | **2** | | | **0** | | |
| **Ethoxyquin** | **0** | **0.04** | | | **0** | | |
