# Supplementary material for: Cellulose Supplementation Early in Life Ameliorates Colitis in Adult Mice
Source: PLoS One. 2013 Feb 20;8(2):e56685. doi: 10.1371/journal.pone.0056685 (PMC3577696; doi:10.1371/journal.pone.0056685)
Supplement: Table S2 — The effects of cellulose supplementation on colonic mucosa associated bacterial families. A. Bacterial families differing with respect to cellulose supplementation [HC: high cellulose vs. LC: low cellulose, control; and 10 days of reversal (HCR10) vs. LC] in our discovery groups. Following 10 days reversal, the microbiome separation decreased between the cellulose supplemented and control groups (7 families differed in HC vs. LC; 4 families differed in HFR10 vs. LC). B. Bacterial families differing in HC and HCR10 group compared to controls in the validation cohort. There were 10 families with significant abundance difference on HC compared to controls. This number decreased to 6 families by 10 days reversal (HCR10 vs. LC). Therefore, similar decreases in differing family numbers occurred in both the discovery and the validation cohorts. Furthermore, on high cellulose diet, bacterial family (Peptostreptococcaceae, Lachnospiraceae, Clostridiaceae increased; Lactobacillaceae and Erysipelotrichaceae decreased in HC group compared to LC) abundances changed the same way in both the discovery and experimental groups (bold). p values represent two tailed non-paired T test, U values represent two tailed non-parametric Mann-Whitney U-test (ns: not significant, na: not applicable). (DOC) [file pone.0056685.s004.doc]

**Table S2. The effects of cellulose supplementation on colonic mucosa associated bacterial families.**

| 1. **Discovery group FAMILY** | | | | | |
| --- | --- | --- | --- | --- | --- |
|  | **LC** | **HC** | **p** | **U** | |
| **Peptostreptococcaceae** | **1.62** | **12.11** | **0.001** | **0.049** | |
| Rikenellaceae | 0 | 0.05 | 0.006 | 0.037 | |
| **Lactobacillaceae** | **21.70** | **9.89** | **0.008** | **0.049** | |
| **Erysipelotrichaceae** | **62.84** | **44.76** | **0.028** | **0.049** | |
| **Lachnospiraceae** | **2.85** | **11.36** | **0.034** | **0.049** | |
| **Clostridiaceae** | **0.36** | **3.65** | **0.036** | **0.049** | |
| Staphylococcaceae | 0.15 | 0.02 | 0.046 | 0.049 | |
|  | **LC** | **HCR10** | **p** | **U** | |
| Verrucomicrobiaceae | 0.02 | 0.29 | 0.007 | 0.046 | |
| Coriobacteriaceae | 0.03 | 0.08 | 0.016 | 0.046 | |
| Lactobacillaceae | 20.68 | 14.35 | 0.039 | 0.049 | |
| Porphyromonadaceae | 0.46 | 0 | 0.054 | 0.037 | |
|  | | | | |  |
| 1. **Validation group FAMILY** | | | | | |
|  | **LC** | **HC** | **p** | **U** | |
| Coriobacteriaceae | 0.365988 | 0.071547 | 0.002391 | 0.0007 | |
| Porphyromonadaceae | 21.71436 | 11.93176 | 0.006233 | 0.0047 | |
| **Lachnospiraceae** | **12.79031** | **23.30765** | **0.011416** | **0.04** | |
| **Peptostreptococcaceae** | **0** | **1.733627** | **2.77E-08** | **na** | |
| Anaeroplasmataceae | 0 | 0.011007 | 0.031863 | na | |
| Bacteroidaceae | 0 | 0.011007 | 0.031863 | na | |
| Bacteroidetes_Other | 8.511282 | 15.67969 | 0.012279 | 0.028 | |
| **Lactobacillaceae** | **4.133187** | **0.836544** | **0.001171** | **0.0013** | |
| Ruminococcaceae | 22.98844 | 32.25647 | 0.011998 | 0.0127 | |
| **Erysipelotrichaceae** | **18.61585** | **3.76995** | **0.008097** | **0.0013** | |
| **Clostridiaceae** | **0.156852** | **1.056687** | **0.001377** | **0.0013** | |
| Lactobacillales_Other | 0.134838 | 0.011007 | 0.002121 | 0.0013 | |
|  | **LC** | **HCR10** | **p** | **U** | |
| Actinomycetales | 0 | 0.027518 | 0.000451 | na | |
| Coriobacteriaceae | 0.365988 | 0.055036 | 0.001153 | 0.0007 | |
| Enterobacteriaceae | 0.002752 | 0.033021 | 0.021613 | ns | |
| Peptostreptococcaceae | 0 | 0.093561 | 0.008005 | na | |
| Firmicutes_Other | 0.533847 | 0.137589 | 0.043775 | 0.0127 | |
| Burkholderiaceae | 0 | 0.022014 | 0.031863 | na | |
| Clostridiaceae | 0.156852 | 1.172262 | 0.047661 | ns | |

**A.** Bacterial families differing with respect to cellulose supplementation [HC: high cellulose vs. LC: low cellulose, control; and 10 days of reversal (HCR10) vs. LC] in our discovery groups. Following 10 days reversal, the microbiome separation decreased between the cellulose supplemented and control groups (7 families differed in HC vs. LC; 4 families differed in HFR10 vs. LC). **B.** Bacterial families differing in HC and HCR10 group compared to controls in the validation cohort. There were 10 families with significant abundance difference on HC compared to controls. This number decreased to 6 families by 10 days reversal (HCR10 vs. LC). Therefore, similar decreases in differing family numbers occurred in both the discovery and the validation cohorts. Furthermore, on high cellulose diet, bacterial family (Peptostreptococcaceae, Lachnospiraceae, Clostridiaceae increased; Lactobacillaceae and Erysipelotrichaceae decreased in HC group compared to LC) abundances changed the same way in both the discovery and experimental groups (bold). p values represent two tailed non-paired T test, U values represent two tailed non-parametric Mann-Whitney U-test (ns: not significant, na: not applicable).
